# From landrace to modern hybrid broccoli: the genomic and morphological domestication syndrome within a diverse *B. oleracea* collection

**DOI:** 10.1038/s41438-020-00375-0

**Published:** 2020-10-01

**Authors:** Zachary Stansell, Thomas Björkman

**Affiliations:** 1 Cornell University, School of Integrative Plant Science, Cornell University, Ithaca, NY 14850 USA; 2 Cornell AgriTech, Cornell University, Geneva, NY 14456 USA

**Keywords:** Plant domestication, Agricultural genetics, Plant breeding

## Abstract

Worldwide, broccoli (*Brassica oleracea* var. *italica*) is among the most economically important, nutritionally rich, and widely-grown vegetable crops. To explore the genomic basis of the dramatic changes in broccoli morphology in the last century, we evaluated 109 broccoli or broccoli/cauliflower intermediates for 24 horticultural traits. Genotype-by-sequencing markers were used to determine four subpopulations within *italica*: Calabrese broccoli landraces and hybrids, sprouting broccoli, and violet cauliflower, and to evaluate between and within group relatedness and diversity. While overall horticultural quality and harvest index of improved hybrid broccoli germplasm has increased by year of cultivar release, this improvement has been accompanied by a considerable reduction in allelic diversity when compared to the larger pool of germplasm. Two landraces are the most likely founding source of modern broccoli hybrids, and within these modern hybrids, we identified 13 reduction-in-diversity genomic regions, 53 selective sweeps, and 30 (>1 Mbp) runs of homozygosity. Landrace accessions collected in southern Italy contained 4.8-fold greater unique alleles per accessions compared to modern hybrids and provide a valuable resource in subsequent improvement efforts. This work broadens the understanding of broccoli germplasm, informs conservation efforts, and enables breeding for complex quality traits and regionally adapted cultivars.

## Introduction

Broccoli (*Brassica oleracea* var. *italica*) and cauliflower (*B. oleracea* var. *botrytis*) are the most widely-grown brassica vegetable crops internationally, with a cumulative production area of 1.4 million Ha^1^. F_1_ hybrid broccoli is the most economically important brassica vegetable crop in the United States with a farm-gate value of ~1 billion USD^2^.

In recent years, considerable progress understanding the *B. oleracea* crop group has been made. Specifically, several key objectives have been accomplished: parsing fundamental genomic architecture^3–5^, publication of high-quality reference genomes^6–8^, evaluating diversity and domestication processes^9–21^, and identifying genomic regions or candidate genes associated with horticultural quality^22–27^ and biotic/abiotic stress resistance^28–35^.

While modern broccoli cultivars are distinct from their landrace precursors in heading induction requirement, time to maturity, crown size and architecture, and secondary metabolic profile^14,17,36–44^, the basis for these dramatic changes remains largely unexplored using genomics era tools, such as genotype-by-sequencing. Here, we build on previous work by clarifying the relationship of elite broccoli germplasm within a larger pool of *italica* germplasm, and characterize the genomic and phenotypic changes that occurred during this improvement process^16^.

The *italica* cultivar group is a member of the **CC** genome *B. oleracea* (2n = 18) coenospecies and was domesticated from crop wild relatives in the Mediterranean Basin by human selection under local conditions, followed by improvement into landrace types in the central Mediterranean region, most likely within the southern Italic Peninsula and Sicily^14,17,19,45–48^. *Italica* domestication is complicated by emergence from a relatively large and admixed pool of landraces, consistent with a Vavilovian model of local assortment of morphologically and physiologically heterogeneous populations^47,49^.

Over time, Calabrese broccoli production and breeding has generally spread westward. Various *italica* and *botrytis* landraces from southern Italy were introduced to the United Kingdom during the 18th century^39^. Although Calabrese broccoli was initially brought to the United States by immigrants from southern Italy, it only gained popularity there post-WWII, following development of improved open-pollinated cultivars such as ‘Waltham 29’ (1950) from the Massachusetts Experiment Station^50,51^. Supported by American and Japanese breeding of commercially successful hybrids such as ‘Premium Crop’ (1975), ‘Packman’ (1983), and ‘Marathon’ (1985), production was shifted to the cooler valleys along the western U.S. coast allowing year-round production^51^. These hybrid breeding efforts increased yield (head size and harvest index), horticultural quality, regional adaptation, while decreasing days to complete growing cycle^39,51,52^. China is now the largest producer of broccoli and Chinese cultivars appear to be derived from a core collection of Japanese germplasm, exhibiting close genetic relationships and reduced diversity^20^.

Traditionally, small farmers and gardeners in periurban farming environments have practiced *in-situ* preservation of diverse and locally adapted *B. oleracea* landraces via seed-saving and informal selection^11,14,17,40,44,53–56^. *Italica* landraces from Italy are more genetically diverse than other *italica* landraces^43^, and this diverse germplasm has been jeopardized, prompting *ex-situ* and *in-situ* conservation efforts and economic sustainability policies^14,17,38,40,44,57^. Several landraces have been suggested as potential *italica* primitives: the highly branching ‘Broccolo Nero’ lacking apical dominance^17^, ‘Mugnoli’ from the Salento region^14,58,59^, and a Sicilian landrace, ‘Cavolo Broccolo Calabrese Tardivo’ that collocated with crop wild relatives^43^.

Several additional *italica* vegetables are known. The sprouting broccoli type is a distinct vegetable and is characterized by many lateral inflorescences, a small apical crown typically bisected by cauline leaves, later heading and flowering, and prized culinary properties.

Purple cauliflower types are most common in southern Italian regions and exhibit an intermediate *italica*/*botrytis* phenotype, with leaf structures more similar to *botrytis*, intermediate developmental arrest stage and glucosinolate profiles, and variable curd coloring (e.g., purple, green, red, or white)^36,38^. The tropical cauliflower type was first bred in 19^th^ century India and is now common in Southeast Asian markets, typically producing cauliflower-like heads above 22 °C and broccoli-like heads below 16 °C^36,60,61^. Violet and tropical cauliflower vegetables appear as *italica*/*botrytis* intermediates in morphologic and population structure analyses^16,29,47^. It is currently unclear if these intermediates exist as recent hybrids or are derived from an ancestral breeding pool. Additional *italica* or *italica*/*botrytis* vegetables are most abundant in southern Italy and include the putative botanical classes Romanesco, Di Jesi, and Maceracta^12,14,17,36^.

To understand and document the improvement process of modern broccoli cultivars from within the larger pool of *italica* germplasm, 109 unique landrace and F_1_ hybrid accessions were evaluated for 24 horticultural quality traits and 31,811 high quality genotype-by-sequencing markers were generated. We applied multiple selection scan methods to contrast a broad sampling of modern broccoli hybrids against a pool of broccoli landraces and identified patterns of population differentiation, regions of reduced diversity, selective-sweeps, runs of homozygosity, and developmental candidates within modern Calabrese broccoli hybrids. When compared with the larger pool of *italica* germplasm, genomic regions enriched in these signatures of crop improvement were considered as possible targets of human selection. This works clarifies the relationship of modern broccoli within *B. oleracea* var. *italica*, describes phenotypic changes that have occurred during improvement, and prepares the foundation for genomics-enabled broccoli improvement.

## Results

**Fig. 1:**
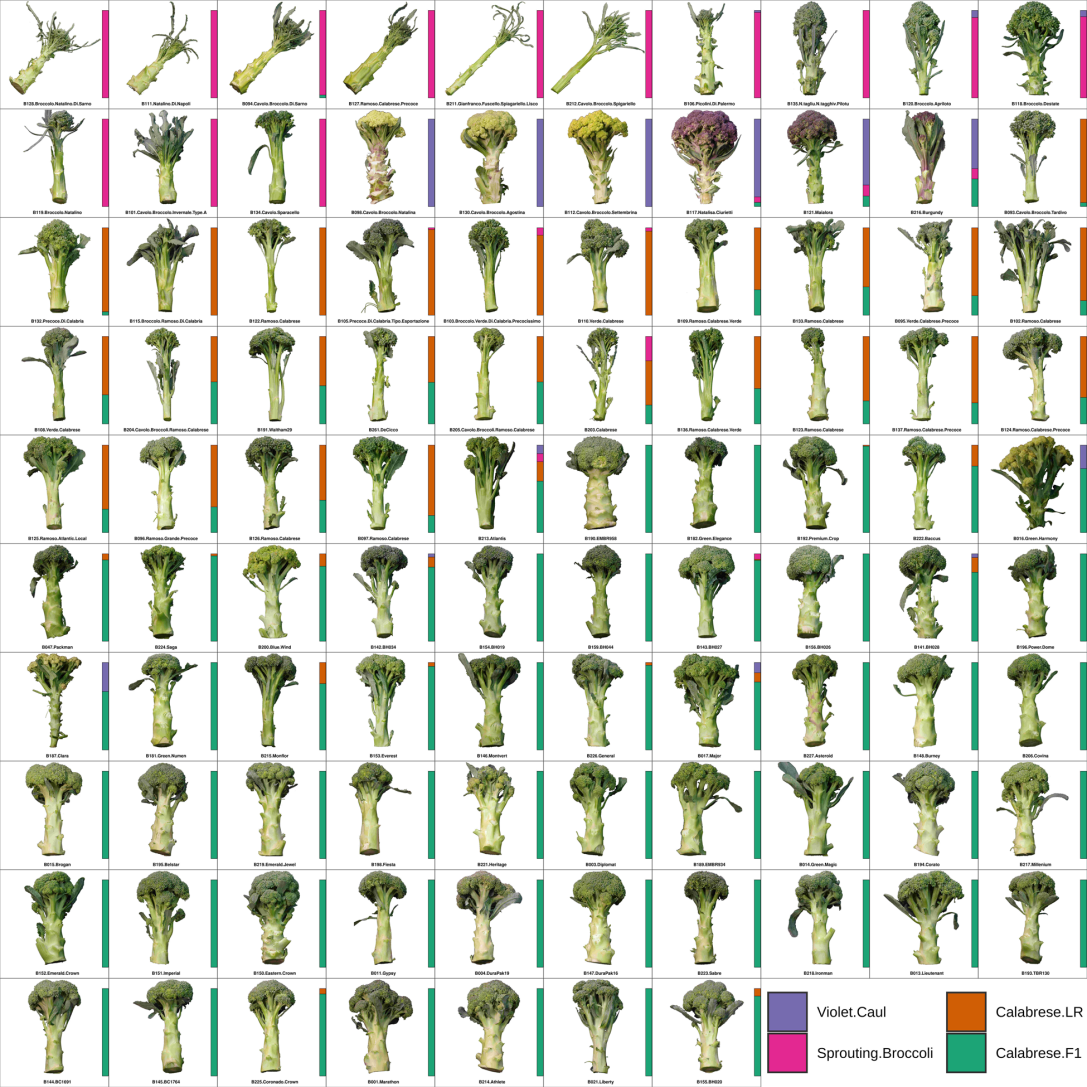
Morphological diversity of *B. oleracea* var. *italica* heading structures. Head images of accessions that produced heading structures in field trials (*N* = 97). All heads were trimmed 16 cm from crown apex and scaled to fit image grid. Inferred population membership generated from structure analysis (*K* = 4; 16,769 linkage pruned SNPs) is presented as vertical bars

Four inferred subpopulations were determined via analysis of genotype-by-sequencing markers of all accessions (*N* = 109; Fig. 1; Supplementary Fig. 1): Calabrese hybrids (*N* = 53), Calabrese landraces (*N* = 28), sprouting broccoli (*N* = 18) and violet cauliflower (*N* = 10), and subpopulation membership was highly concordant with phenotypic evaluations within replicated field trials.

### Genotyping

**Fig. 2:**
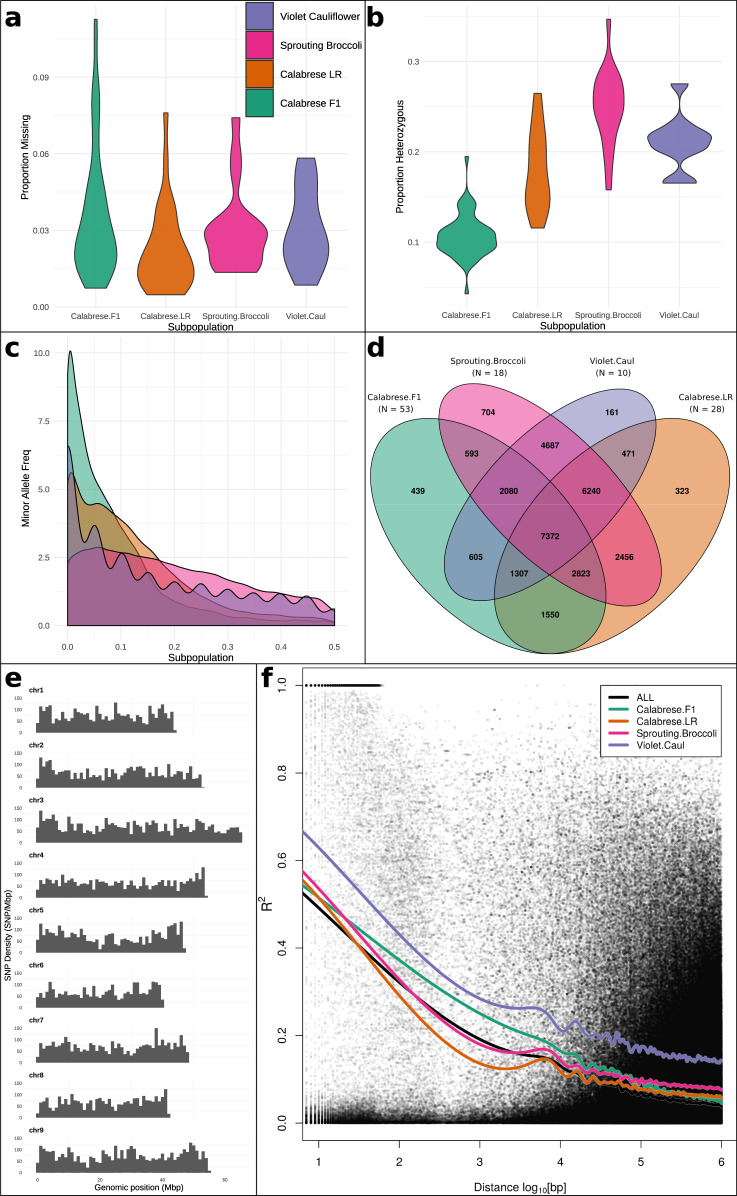
GBS summary statistics for 31,811 high-quality SNP markers. **a** Proportion of site missingness across all markers by subpopulation. **b** Proportion of site heterozygousity across all markers by subpopulation. **c** Pooled minor (second most common) allele frequency by subpopulation. **d** Unique polymorphic SNPs by subpopulation. **e** SNP density by chromosome (chr1-chr9) and genomic position in 1 Mbp windows (*x*-axis). **f** Linkage disequilibrium decay plotted as pairwise r^2^ against log_10_(bp) with subpopulation data fitted with cubic smoothing spline (spar = 0.5)

To distinguish among closely related genotypes and understand genomic variation, we conducted genotype-by-sequencing of all evaluated accessions, producing 1,185,484,626 raw reads, with over 88% of bases surpassing Q30. The raw reads generated 10,108,099 tags producing 247,220 aligned SNPs. After filtering, 31,811 high quality SNPs were retained for further analysis. Averaged across these SNPs, site missingness and heterozygosity was 3.1% and 15.9%, and minor allele frequency was 12.8% (Fig. 2a–d). SNPs per chromosomes ranged from 2867 (chr8) to 4662 (chr3) and SNP density per chromosome (SNPs/Mbp) ranged from 54.3 (chr4) to 66.2 (chr9) (Fig. 2e).

Of the 31,811 polymorphic markers, 7372 (23.2%) were shared among all supopulations (Fig. 2d). By subpopulation, Calabrese hybrid accessions contained the fewest unique polymorphic alleles (8.3 accession^−1^) and sprouting broccoli types contained the most (39.1 accession^−1^). When comparing the polymorphic markers between the inferred Calabrese hybrid and Calabrese landrace subpopulations, 49.7% were common to both (Calabrese hybrid ∪ Calabrese landrace = 26,259; Calabrese hybrid ∩ Calabrese landrace = 13,052; Calabrese landrace – Calabrese hybrid = 9,490), and Calabrese landraces contained 4.8-fold greater unique alleles per accession compared to Calabrese hybrid accessions.

When comparing the inferred Calabrese hybrid subpopulation against all other taxa, site missingness was not significantly higher (Fig. 2a), although heterozygousity was reduced in the same comparison (Fig. 2b; *p* < 0.01). We observed a right skew and an increase in minor allele variants in Calabrese hybrids compared to other *italica* types (*p* < 0.01). For all accessions pooled across all markers, linkage disequilibrium decayed rapidly to background levels (*r*^2^ < 0.2) by 0.82 kbp (Fig. 2f). Linkage decay to background levels was substantially different by subpopulation, decaying fastest in the Calabrese landraces (0.30 kbp), followed by sprouting broccoli (0.63 kbp), Calabrese hybrids (4.12 kbp), and violet cauliflower (52.1 kbp).

Of the SNP variants identified between markers and the reference genome, 4,523 were predicted to result in missense, nonsense (129), and silent (5,195) amino acid changes [Supplementary Data 1 (variants.ods)]. In several agricultural crops, modern accessions exhibit AT-bias across polymorphic sites compared to their respective landraces^62^, and this finding was confirmed when comparing modern Calabrese broccoli hybrids against the less improved *italica* germplasm. Mean genome-wide [AT] base composition was highest in the Calabrese hybrid subpopulation and different between subpopulations (*p* < 0.01; sprouting broccoli = 0.312 < violet cauliflower = 0.333 < Calabrese landraces = 0.344 < Calabrese hybrids = 0.373).

### Diversity analysis

#### Principal components

**Fig. 3:**
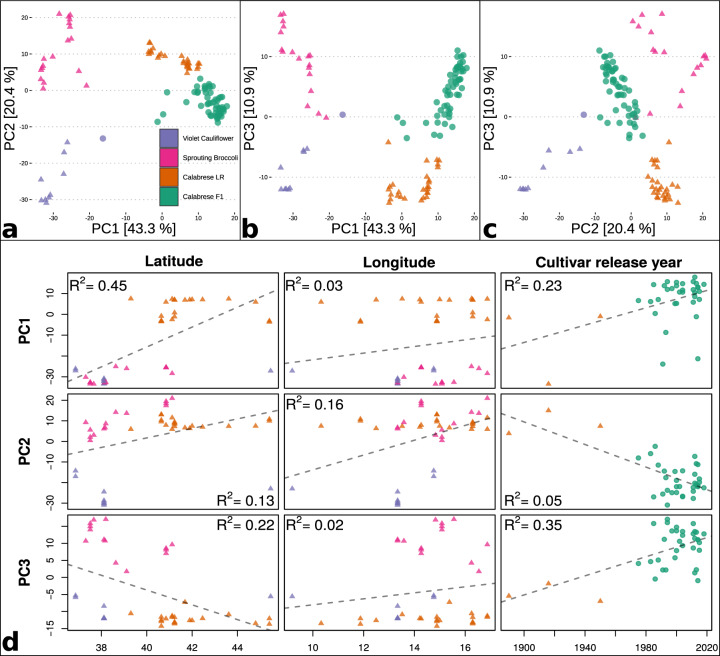
Patterns of population structure by principal component analysis of accessions using 31,811 SNP markers with colors assigned by inferred subpopulation (green = Calabrese hybrids, orange = Calabrese landraces, pink = sprouting broccoli, purple = violet cauliflower, landrace = triangles, hybrid = circles). **a–c** Pairwise comparisons between multidimensional scaling axes of PC axes 1–3 with percent variance explained printed along axes. **d** Nonstratified linear model comparing PC axes 1–3 [rows] against accessions with geographical provenances [latitude (°N), longitude (°W), and cultivar release year; columns] with model *R*^2^ printed in subplots

The genetic diversity, phylogeny, and population structure of 109 distinct *B. oleracea* accessions was evaluated using 31,811 genome-wide markers. Principal component analysis (PCA) using these markers effectively resolved the 109 accessions into four subpopulations and the first three axes explained a cumulative 74.6% of model variation (Fig. 3a–c). The Calabrese accessions were clearly resolved from other accessions, and PCA axis 3 formed a gradient between the Calabrese subpopulations, with early modern open-pollinated Calabrese accessions B261.DeCicco and B191.Waltham29 located between the landrace and hybrid subpopulations, and recently released F_1_ hybrids were located at the extremity. The tropical cauliflowers B187.Clara and B016.Green.Harmony and the violet cauliflower F_1_ hybrid B216.Burgundy was located between the Calabrese hybrid and violet cauliflower groups, consistent with an admixed breeding pedigree. Calabrese landraces were collected along the entire Italic Peninsula, whereas the sprouting broccoli and violet cauliflower accessions were collected nearly exclusively in the Southern Italic Peninsula and Sicily and PC axis 1 versus collection latitude exhibited the strongest association with collection location (*R*^2^ = 0.45) (Fig. 3d). PC axis 2 was most correlated with cultivar release year (*R*^2^ = 0.50). SNP coefficients explaining PCA variance ranged from −0.027 to +0.050, and the top 1% PC coefficients by absolute value were retained for further analysis. Of these high-loading SNPs, 762 (41.6%) were located within gene intervals, and 13 (Bo1g021960, Bo1g039650, Bo1g051570, Bo2g018320, Bo2g041560, Bo3g001090, Bo3g080160, Bo3g094030, Bo4g045930, Bo5g010600, Bo5g150300, Bo6g080130, Bo7g088960) were marked as high impact variants [Supplementary Data 1 (variants.ods)].

### Phylogeny, identify-by-state, and structure

**Fig. 4:**
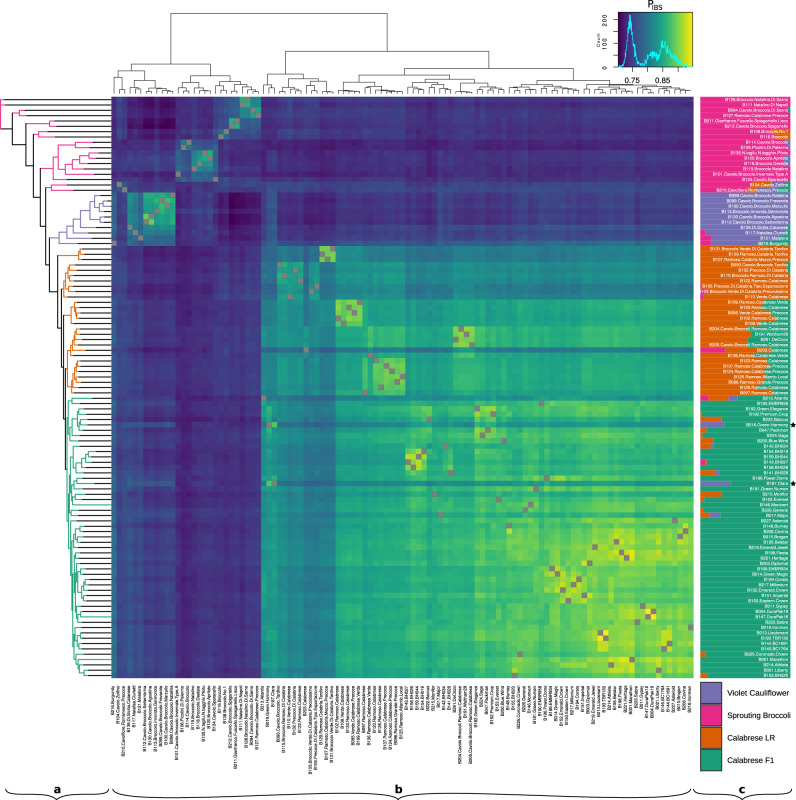
Three analyses of relatedness of all evaluated accessions (*N* = 109). **a** Maximum-likelihood based phylogenetic inference using 31,811 SNPs (GTR + GAMMA substitution model, *N* = 100), indicating four subpopulations (legend bottom right; violet cauliflower = purple, sprouting broccoli = pink, Calabrese broccoli landraces = orange, Calabrese broccoli hybrids = green). **b** Similarity matrix of all accessions calculated as probability of identity by state, averaged over all non-missing loci (legend top; range 0.70–0.95), indicating genetic similarities between accessions and inferred subpopulations. Similarity matrix rows are sorted by RAxML phylogenetic inference order and columns are sorted by genetic similarity between genotypes. **c** Proportion of subpopulation membership of accessions within via FastStructure analysis using 16,341 linkage pruned SNPs (*K* = 4; legend bottom right). Rows are sorted by phylogenetic inference, and tropical cauliflower accessions indicated by (*)

Phylogenetic analysis identified four main subpopulations (Fig. 4a, Supplementary Fig. 2). All Calabrese accessions formed a monophyletic clade, and the Calabrese hybrids formed a monophyletic clade within the Calabrese clade. The violet cauliflower accessions and the sprouting broccoli subpopulations formed monophyletic and a paraphyletic clades, respectively.

Identity by state analysis was used to construct a similarity matrix using all lines (Fig. 4b). Mean probability of identity by state (P_IBS_) was 0.80 across all lines and pairwise P_IBS_ ranged from 0.70 (B212.Cavolo.Broccolo.Spigariello versus B100.Cavolo.Broccolo.Marzullo) to 0.95 (B193.TBR130 versus B013.Lieutenant). When comparing individual accessions to all other accessions, the most distinct and similar accessions were B106.Picolini.Di.Palermo (mean *P*_IBS_ = 0.73) and B001.Marathon (mean *P*_IBS_ = 0.84). Two groups of Calabrese landraces were very similar (*P*_IBS_ > 0.90): the nonheading accessions (B107.Ramoso.Calabria.Mezzo.Precoce, B129.Ramoso.Calabria.Tardivo, and B131.Broccolo.Verde.Di.Calabria.Tardivo) and four small-headed Calabrese accessions (B095.Verde.Calabrese.Precoce, B102.Ramoso.Calabrese, B108.Verde.Calabrese, B109.Ramoso.Calabrese.Verde, and B133.Ramoso.Calabrese). Several early commercial landraces (B191.Waltham29, B204.Cavolo.Broccoli.Ramoso.Calabrese, B205.Cavolo.Broccoli.Ramoso.Calabrese, and B261.DeCicco) and accessions from the USDA-USVL breeding program (B143.BH027, B154.BH019, B156.BH026, and B159.BH044) appeared highly related (*P*_IBS_ > 0.90).

Several interesting patterns of relatedness were observed in population structure analysis (Fig. 4c). Members of the green population were exclusively Calabrese F_1_ hybrids (mean membership = 95.1%; range = 58.9–100.0%). Majority members of the orange population were exclusively identified as Calabrese landraces (*N* = 28; mean membership = 77.6%; range = 50.6–100.0%). The pink majority subpopulation was comprised entirely of accessions collected from the Southern Italic Peninsula and Sicily (*N* = 18; mean = 91.4%; range = 52.0–100.0%). Majority members of the purple subpopulation (*N* = 10; mean membership = 91.7%; range = 56.7–100%) was comprised entirely of purple cauliflower types that were characterized by an intermediate *italica* and *botrytis* phenotype, a purple to off-white heading inflorescence, intermediate meristem arrest stage, long connected petiole wings, and little to no lateral shoot formation.

Calabrese landraces were partially admixed with the Calabrese hybrid subpopulation (mean = 20.9%; range = 0.0–48.1%), and some Calabrese hybrids were partially admixed with the Calabrese landrace subpopulation (mean membership = 3.0%; range = 0.0–24.1%), including the accessions B215.Monflor (24.1%), ‘Packman’ related B222.Baccus (23.9%), and the *alboglabra* x *italica* hybrid B213.Atlantis (22.2%). Members of the sprouting broccoli and violet cauliflower subpopulations shared very little membership with the Calabrese hybrid (mean = 1.2% and 5.2%) or Calabrese landrace subpopulations (mean = 3.9% and 0.0%).

The sprouting broccoli and violet cauliflower subpopulations have experienced very little admixutre with each other or Calabrese types. The sprouting broccoli (pink) subpopulation component was almost never identified within other subpopulations (Calabrese hybrid = 0.0%, Calabrese landrace = 1.5%, violet cauliflower = 0.0%), and the violet cauliflower subpopulation (purple) was uncommon in members of other subpopulations (Calabrese hybrid = 1.7%, Calabrese landrace = 0.0%, sprouting broccoli = 3.4%).

However, several *italica*/*botrytis* phenotypic intermediates were confirmed in structure analysis: the accession B216.Burgundy is a F_1_ hybrid with an *italica x botrytis* pedigree (56.7% purple and 31.6% green). Two Calabrese hybrid majority members are commercial tropical cauliflower hybrids with temperature-sensitive heading structure and contained partial admixture within the purple subpopulation (B016.Green.Harmony = 26.8%, B187.Clara = 33.2%).

### F_st_, reduction in diversity, selective sweeps, and runs of homozygosity

**Fig. 5:**
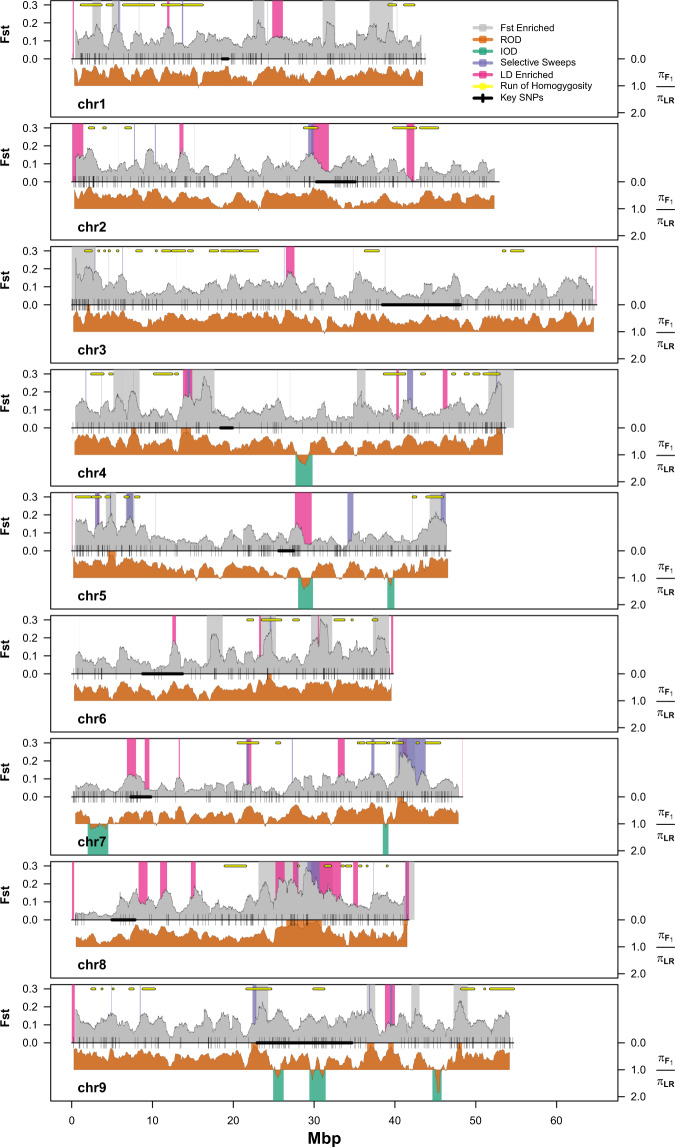
Genome-wide scans of selection and domestication footprints when comparing Calabrese hybrids with *italica* landraces using 31,811 SNPs markers by chromosome (chr1-chr9) and physical position (Mbp; *x*-axis). Fixation index [F_st_; gray trace; left *y*-axis] and ratio of nucleotide diversity [orange trace; right *y*-axis] comparing Calabrese hybrids against all other accessions, with enriched F_st_ regions printed in gray boxes, with top reduced (ROD=orange) and enriched (IOD=green) nucleotide diversity regions printed as boxes. Selective sweeps (purple) and linkage disequilibrium enriched (pink) regions are printed as boxes. Pooled runs of homozygosity identified within the Calabrese hybrids are printed as yellow bars. Key PCA loading SNPs and centromeric regions derived from half-tetrad analysis^6^ are printed as black ticks and bold centerlines, respectively

Several notable genomic patterns were revealed when comparing the Calabrese hybrid subpopulation against all other accessions (Fig. 5). Fixation index (F_st_) is a measure of structure related population differences. Genome-wide scans comparing Calabrese hybrids against all other accessions identified 24 genome-wide F_st_ enriched regions in modern Calabrese hybrid germplasm [Supplementary Data 2,.(genomic-regions.ods)]. Pooled weighted F_st_ between subpopulations were moderately low between Calabrese landraces and hybrids (*F*_st_ = 0.09), and very high between Calabrese hybrids and violet cauliflower (*F*_st_ = 0.33) and sprouting broccoli (*F*_st_ = 0.26). Calabrese landraces were less differentiated between the violet cauliflower (*F*_st_ = 0.25) and sprouting broccoli (*F*_st_ = 0.16) subpopulations. The sprouting broccoli and violet cauliflower populations were moderately differentiated (*F*_st_ = 0.14). Fifteen regions (Fig. 5 [gray]) with elevated F_st_ were identified and contained 4208 genes. Eight of these elevated F_st_ region genes were identified as high impact variants (Bo1g078320, Bo3g001090, Bo4g196000, Bo5g014760, Bo6g079470, Bo6g080130, Bo6g099010, Bo8g088460) [Supplementary Data 3 (genomic-regions.ods)].

Selection driven sweeps can result in reduced genetic diversity in improved germplasm compared to undomesticated or landraces germplasm. Overall, Calabrese hybrids experienced considerable genome-wide reduction in nucleotide diversity when compared to all other accessions (ROD = 0.55; Fig. 5 [orange]). In this comparison, 13 reduced and 8 enriched nucleotide diversity genomic regions were identified (Fig. 5) spanning on average 1.0 Mbp (range = 0.4–4.4 Mbp) and 1.57 Mbp (range = 0.7–2.6 Mbp), respectively. Some of the 13 regions were remarkably reduced in nucleotide diversity; for example, the chr8:29.8–30.6 Mbp region, ROD was reduced to 0.05. Of the 8 enriched diversity regions in the Calabrese hybrid subpopulation, three were identified on chromosome 9. The most notable enriched diversity region (ROD = 1.87) was located near the end of chromosome 9 (chr9:44.6–45.8 Mb). Of the 1906 and 1174 genes contained in the reduced and enriched intervals, three reduced (Bo5g014760, Bo6g080130, Bo8g088460) and two enriched (Bo5g126850 and Bo7g009560) SNPs were designated as high impact variants.

Analysis of the Calabrese hybrid subpopulation identified 53 high-likelihood selective-sweep intervals spanning from 0.1–3.4 Mbp (likelihood = 9.3 to 17.3; Fig. 5 [purple]). These intervals contained 1861 genes, and six were classified as high-impact variants (Bo1g017170, Bo5g113490, Bo5g113510, Bo5g148470, Bo5g150300, Bo8g088460).

Runs of homozygousity are predictors of whole-genome inbreeding, and longer runs are evidence of more recent selective pressure^63^. When scanning all accessions, we identified 88 (>1 Mbp) runs of homozygosity (Fig. 5 [yellow]). These regions were nearly exclusively comprised of Calabrese hybrid accessions and 11.4% of these regions included 10 or more Calabrese hybrid accessions.

We detected four unusually feature-rich genomic regions (chr4:14.3–14.6; chr7:40.9–41.1; chr8:27.9–30.7, and chr8:41.4–41.5) that exhibited evidence of selective sweeps, elevated F_st_, high LD, and high ROD. Of the 24 elevated F_st_ regions, there were 18 instances of a selective-sweep (75.0%) and 16 instances (66.7%) of a run of homozygousity collocating with a given F_st_ region. Selective sweeps were never identified within enriched diversity regions, but were identified in 12 of the 13 reduction in diversity regions. The only reduced diversity region without a selective sweep was chr9:47.7–48.3 Mbp, adjacent to the strongest enriched diversity region (chr9:44.6–45.8 Mb).

### Phenotyping

**Fig. 6:**
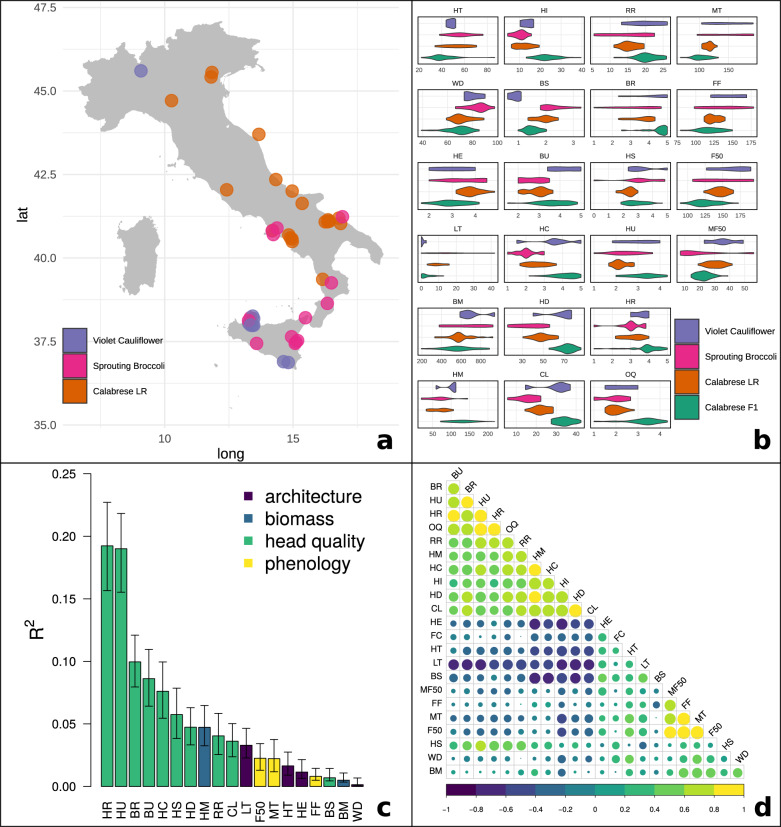
Phenotypic summary of accessions evaluated in field trials. **a** Collection locations Italian landraces with provenances. Color indicates inferred subpopulation membership derived from structure analysis. Jitter is added to points to distinguish accessions collected at the same location. **b** Trait distribution disaggregated by subpopulation (Supplementary Table 2 for units). **c** Proportional contribution of traits to overall quality (OQ) by relative importance analysis using 1000 bootstraps of the lmg algorithm. **d** Spearman correlation matrix between all evaluated traits; positive and negative correlations are printed in yellow and blue, with circle size scaled to magnitude

To capture the diversity contained within *italica* germplasm, seed from 54 commercial F_1_ hybrid broccoli and 55 landrace/open-pollinated accessions was collected from as many breeding programs and distribution channels as possible: Asgrow (2), Bejo (5), Emerald (2), Enza-Zaden (1), Evergrow (2), the USDA Agricultural Research Service (7), Harris-Moran (1), Johnny’s Seed (4), Known-You Seed Company (2), Northeast Seed (1), Peto Seed (2), Sakata (10), Seminis/Vaden Bosch/Royal Sluis (8), Syngenta (4), Tainong (2), and Takii (1) (Supplementary Table 1, Fig. 1). Of the 47 landrace accessions collected in Italy, 43 contained geographic provenances (range 36.86–45.46 °N and 9.19–16.87 °E; Fig. 6a).

In total, 24 horticultural quality traits within four trait categories were evaluated within two trial environments (Supplementary Table 2, Fig. 6b–d, Supplementary Data 3 [pheno.csv]) to compare morphological changes of modern Calabrese broccoli hybrids against the Calabrese landraces and the larger pool of *italica* diversity. To identify trends in improvement, traits were regressed against the year of cultivar release.

#### Architectural

Plant size at maturity is an important yield characteristic, with more compact plants allowing higher density plantings. The architectural traits plant height and width ranged from 22.0 (B189) to 86.4 (B213) cm tall and 38.9 (B190) to 97.8 (B111) cm wide and were different between inferred subpopulations (*p* < 0.01). The Calabrese hybrid subpopulation was the shortest and narrowest (41.0 × 68.9 cm). Plant height and width was not correlated with cultivar release year. Higher head extension above leaf rosette enables efficient harvest and was different between subpopulations (*p* < 0.01) and negatively correlated with heading quality (*r*_s_ = −0.32), harvest index (*r*_s_ = −0.65), cultivar release year (*p* = 0.02). Calabrese landrace accessions exhibited greater head extension (3.83) when compared to Calabrese hybrid accessions (3.02; *p* < 0.01).

A single central head is a primary goal in broccoli breeding programs as lateral shoots increase harvest difficulty. The number of lateral shoots per plant ranged from 0.0 (ten accessions) to 42 (B211) and was negatively correlated overall quality (r_s_ = −0.57) and cultivar release year (*p* < 0.01). The Calabrese hybrid subpopulation exhibited fewer lateral shoots (3.0) than Calabrese landraces (9.4) and sprouting broccoli (16.8), but more than violet cauliflower (0.6).

#### Biomass

While above-ground plant biomass has not increased over time, head mass and harvest index has, consistent with the breeding objective of enabling denser plantings by producing larger and heavy heads from compact plants. All biomass traits evaluated were different between inferred subpopulation (*p* < 0.01). Above-ground biomass ranged from 193.1 (B187) to 941.9 g (B098) and head mass ranged from 18.8 (B212) to 218.4 g (B190). Calabrese hybrid heads were heavier (128.8 g) than Calabrese landraces (68.1 g), sprouting broccoli (66.7 g), and violet cauliflower (98.3 g) heads. Head mass was positively associated with cultivar release year (*p* < 0.01) with a slope of +0.89 g year^−1^. Harvest index ranged from 4.2% to (B211) to 39.6% (B187).

Harvest index was positively correlated cultivar release year (*p* < 0.01) and mean Calabrese hybrid harvest index (23.2%) was nearly double that of Calabrese landrace landraces (12.0%), indicative of strong selection pressure during improvement.

#### Head quality

Improved head quality is a primary target improvement and is required for high quality, regionally adapted cultivars. Average bead size at harvest ranged from to 0.6 mm (B130) to 3.28 mm (B094) and was negatively associated with cultivar release year (*p* < 0.01). The inferred subpopulations differed in bead size, and modern broccoli hybrids (Calabrese hybrid = 1.50 mm) have smaller beads than broccoli landraces (Calabrese landrace = 1.92 mm). The violet cauliflower subpopulation exhibited the highest flower bud uniformity (4.2) compared to the Calabrese hybrid (3.6), Calabrese landrace (2.9), and sprouting broccoli (2.7) subpopulations, and bud uniformity was positively correlated with cultivar release year (*p* = 0.023).

The head mass characteristics: head compactness, head diameter, average cluster width, and first rank branching were all different between subpopulation (*p* < 0.01). Head compactness was positively associated with cultivar release year (*p* < 0.01), ranging from 2.0 (sprouting broccoli) to 4.1 (Calabrese hybrids). Head diameter per accession ranged from 16.1 mm (B211) to 86.0 mm (B004) and was positively associated with cultivar year release (*p* < 0.01). Between subpopulations, head diameter ranged from 37.0 mm (sprouting broccoli) to 72.6 mm (Calabrese hybrids) and Calabrese hybrid head diameter was 23.6 mm greater compared to the Calabrese landrace accessions. Average cluster width and first rank branching were correlated with cultivar release year (*p* < 0.01). Per subpopulation, cluster width and first rank branching ranged from 15.9 (sprouting broccoli) to 34.1 mm (Calabrese hybrid) for cluster width and 15.2 (sprouting broccoli) to 20.6 mm (Calabrese hybrid) for first rank branching.

The presence of cauline leaves bisecting the heading structure at maturity is a common quality flaw in heat sensitive broccoli cultivars. Bracting suppression ranged from complete (5.0; B004) to none (1.0; B101) but was not significantly associated with cultivar release year. Bracting suppression was different between subpopulations (*p* < 0.01) and stronger in Calabrese hybrid (4.5) compared to Calabrese landrace accessions (3.6).

A convex and uniform crown shape is required for high quality broccoli production and helps to shed water during the pre-harvest interval. Head morphology traits were different between inferred subpopulations (*p* < 0.01), but were not strongly associated with cultivar release year. Between subpopulations, head shape ranged from 2.4 (Calabrese landrace) to 3.3 (violet cauliflower), and head uniformity ranged from 2.2 (Calabrese landrace) to 3.3 (Calabrese hybrid).

Breeding efforts to expand the adapted range of broccoli have focused on decreasing the minimum chilling hour requirements for normal heading and increasing overall heat tolerance. Sensitive hybrids experience flaws at the meristem arrest and proliferation stages, resulting in a failure to produce heads, irregular flower bud sizes, and other defects. Heat tolerance ranged from none (1.0; B101) to very high (5.0; B141) and was strongly correlated with overall quality (*R*^2^ = 0.78), but not with cultivar release year. On average, the Calabrese hybrid subpopulation (3.8) exhibited greater heat tolerance (*p* < 0.01) compared to the Calabrese landrace subpopulation (3.1).

Overall quality, an aggregate measurement of horticultural quality within a given environment has been used to select optimal hybrids in breeding trials^52^. Here, overall quality ranged from very low (1.0; B101) to very high (4.33; B206) and was associated with cultivar release year (*p* = 0.02). Per inferred subpopulation, overall quality was different (*p* < 0.01), ranging from 1.9 (sprouting broccoli) to 3.2 (Calabrese hybrid). Calabrese hybrid accessions performed +1.2 points better than Calabrese landrace accessions (*p* < 0.01). Relative importance analysis indicated that variation in heat response (19.0%) and head uniformity (19.0%) explained the greatest amount of variation in overall heading quality (OQ) and the remaining modeled traits cumulatively explained less than 16.4% of variation in overall quality (Fig. 6c).

Flower color was predominately yellow, although six sprouting broccoli accessions produced white flowers (B118, B119, B120, B135, B211, and B212).

#### Phenology

All of the phenology traits were strongly correlated between each other, different between subpopulations, and not correlated with cultivar release year. Days to from sowing to head maturity ranged from 67.5 (B187) to 193 d (B094 and B128), with 16 accessions failing to consistently head. Calabrese hybrid types reached head maturity the fastest (103.8 d) and the sprouting broccoli types the slowest (152.7 d). Days to first flowering ranged from 75.3 (B016) to 178.5 d (B127) and 21 lines did not achieve any flowering. Calabrese hybrid accessions flowered the earliest (119.4 d) and the violet cauliflower the latest (147.2 d). Days to 50% flowering ranged from 89.7 (B016) to 194 (B119) and 22 lines did not achieve 50% flowering. Days from head maturity to 50% flowering ranged from 7.0 (B127) to 56.0 (B119), although B119 and several other landrace accessions exhibited highly heterogeneous within-plot heading and flowering that likely inflated this value. By subpopulation, days from head maturity to 50% flowering varied from 21.5 (sprouting broccoli) to 35.7 d (violet cauliflower).

## Discussion

To our knowledge, this study provides the most comprehensive investigation of the morphological and genomic diversity of *B. oleracea* var. *italica* germplasm. Using genotype-by-sequencing, we generated 31,811 high-quality SNP markers with a mean density of 65.2 SNPs/Mbp which is suitable for subsequent GWA studies given genome-wide linkage disequilibrium. These markers were used to infer four subpopulations by evaluating principal components, phylogeny, identity-by-state, population structure. We then evaluated critical genomic regions and targets of selection in modern F_1_ Calabrese broccoli by evaluating genome-wide population differentiation, reduced or enriched nucleotide diversity, selective-sweeps, and runs of homozygosity (Figs. 2–4). These 109 unique landrace and F_1_ accessions were phenotyped for 24 horticultural traits within two growth environments (Figs. 1, 6b, c).

Modern F_1_ hybrid Calabrese broccoli was delineated as a monophyletic clade within a larger monophyletic clade of Calabrese broccoli and was clearly differentiated by principal component and structure analysis. We observed lower heterozygosity in the Calabrese F_1_ hybrids compared to the other inferred subpopulations, a somewhat unexpected result given that F_1_ hybrids crossing schemes typically aim to increase heterozygosity. This result is the most consistent with repeated crossing within a pool of highly homogeneous parental germplasm used to generate Calabrese F_1_ hybrids. Our results are consistent with a reduction in genetic diversity that occurred during breeding efforts in the last century, eroding the genetic base of Calabrese broccoli germplasm. Individually and as a group, hybrid Calabrese broccoli accessions were more homozygous, had greater within-group similarity, and exhibited considerably reduced allelic diversity (Figs. 2d and 4a–c). In addition, there was a positive [AT] nucleotide composition bias in hybrid Calabrese accessions, concordant with results previously identified in elite maize and soybean cultivars^62^.

We expected to identify selective sweeps in the vicinity of genes that regulate horticultural quality traits under selection during domestication and further crop improvement. Several key genomic regions bear strong signals of domestication and selection, and warrant further investigation. This terminal chromosome 9 region has been shown to harbor homologs of flowering and chilling requirement genes *TLF2*, *COL1*, *CO*, and *FLC*^32,64–67^ and has been linked to temperature-dependent time to curd initiation in a double-haploid cauliflower^68^, heat-tolerance in a double-haploid *italica* mapping population^69^, and a head forming and later flowering time phenotype in a double-haploid *alboglabra* x *italica* mapping population^27^. We identified a strong F_st_ region (chr9:47.4–48.9) that contained 236 *A. thaliana* homologs including *CO* (Bo9g163730) and *COL1* (Bo9g163720). Interestingly, this region was flanked by the strongest genome-wide increase in diversity region (chr9:44.6–45.8 Mb). A region of increased diversity flanking a decreased diversity region would be expected given the casual gene in the decreased diversity region was a repeated target for backcrossing. These results support previous work that the chromosome 9 50 Mb region is a likely target for domestication and improvement in Calabrese broccoli. In Calabrese broccoli hybrids, the largest and strongest region of population differentiation was a 7.5 Mbp region (chr8:23.4–30.9) that collocated with a region of 95% reduced nucleotide diversity (chr8:26.6–31.0 Mb), three linkage disequilibrium enriched blocks, three selective sweeps intervals, and was flanked by several runs of homozygosity. This region contained 26 of the top 1% SNPs associated with variation in PCA population differentiation.

Calabrese hybrids exhibited superior horticultural quality characteristics compared to other *italica* types, including Calabrese landraces. Unlike many F_1_ hybrid crops when compared to their respective landraces, Calabrese broccoli hybrids did not necessarily produce larger plants, rather producing larger, heavier, more compact and uniform heads, which comprised a larger fraction of total above-ground biomass. When comparing head mass by year of cultivar release, on average, breeding efforts in the last 130 years have increased total head mass by ~0.9 g per year. Modern hybrid Calabrese broccoli heads exhibited greater bracting suppression and more uniform flower buds when compared to Calabrese broccoli landraces. The linkage of lower head extension with other quality traits could result from hitchhiking or as a pleiotropic effect resulting from selection for stronger meristem arrest.

Although no collected Calabrese broccoli landraces were collected in Sicily, they were collected throughout the Italic Peninsula, and not exclusively in Calabria (Fig. 6a). These accessions are clearly morphologically recognizable as Calabrese broccoli, although they exhibited slower head maturity and flowering and inferior heading qualities compared to modern hybrid Calabrese broccoli. Two historically important commercial open-pollinated accessions, B261.DeCicco (1890) and B191.Waltham29 (1950), were partially admixed in structure analysis with modern hybrid Calabrese broccoli, and this relationship was confirmed by principal component analysis. These historical commercial types share close genetic similarity to other Calabrese landrace accessions such as B204.Cavolo.Broccoli.Ramoso.Calabrese and B205.Cavolo.Broccoli.Ramoso.Calabrese. These accessions are, or are closely related to, the founding germplasm exploited during the initial breeding of modern Calabrese hybrids.

Sprouting broccoli accessions were collected either in Sicily or the southern Italian Peninsula below 41 °N and exhibited fewer signals of selection and domestication compared to other broccoli types. The sprouting broccoli morphotype was characterized by large plants with many lateral shoots, long but narrow leaf blade outlines, and smaller heads that lacked apical dominance and suppression of bracting. Notably, members of this subpopulation were also far more variable for many horticultural quality traits when compared with Calabrese broccoli, especially for head quality (first rank branching, bract suppression, head extension) and phenology traits (days to maturity and flowering), and lateral shoot formation. One-third of the sprouting broccoli accessions produced white flowers, a trait not observed in any other inferred subpopulation. The morphological diversity of these sprouting broccoli types is mirrored genetically; sprouting broccoli accessions were far more rich in unique polymorphic alleles per accession when compared to Calabrese landraces and hybrids. While the head morphology of some sprouting broccoli accessions (e.g.; B119 and B134) resembled the Calabrese broccoli ideotype, these accessions were clearly genetically resolved from Calabrese broccoli and may be an example of a parallel or convergent domestication syndrome within *italica*.

We did not observe clear evidence that sprouting broccoli is a direct recent progenitor of Calabrese broccoli or violet cauliflower. In fact, the genetic distance between sprouting broccoli and Calabrese broccoli is roughly the same as the distance between Calabrese broccoli and cauliflower^16^, raising the question of the placement of sprouting broccoli within *B. oleracea*. The distinctions between the Calabrese and sprouting broccoli types may be explained by reproductive isolation due to either geographic isolation (Sicily vs. the Italic Peninsula) or *in-situ* cultural practices isolating these groups as distinct crops. In a separate analysis that derived SNPs generated from alignment to a different *B. oleracea* reference genome^8^, the members of the sprouting broccoli subpopulation were differentiated into separate supopulations, and these groups were morphologically resolved by differences in head mass, head shape, and bracting suppression, indicative of further structure within the sprouting broccoli subpopulation. Interestingly, many of the sprouting broccoli accessions bear some morphological similarities to Chinese kale (*B. oleracea* var. *alboglabra*), such as a weaker apical dominance, smaller, highly bracted heads with large flower buds, and variable white/yellow flower color, although this relationship cannot be evaluated here. Overall, the sprouting broccoli likely represents a valuable pool of genetic diversity that may prove useful for Calabrese improvement efforts as a source of disease-resistance alleles or horticultural quality characteristics.

Collected landraces assigned to the violet cauliflower subpopulation were collected almost entirely in Sicily (except for B139.Di.Sicilia.Catanese, a likely import from Catania). The violet cauliflower population was similar to other *botrytis* types, overall exhibiting an earlier meristem arrest stage compared with sprouting and Calabrese broccoli accessions, although arrest stage ranged from floral primordia to fully developed flower buds. Under unfavorable environmental conditions, Calabrese broccoli hybrids often produce loose heads with irregular bud uniformity. In this evaluation, some violet cauliflower accessions consistently exhibited large, uniform, and compact heading structures and high bud uniformity, traits potentially useful in Calabrese broccoli breeding programs. It has been previously proposed that curding type *botrytis* arose from heading Calabrese broccoli via intermediate Sicilian types^12,47^. Evaluating the accessions observed in this study, it is unlikely that the Calabrese landraces formed the genetic basis of the violet cauliflower accessions. With the exception of recent intentional *italica*/*botrytis* hybrids, these subpopulations were clearly distinct and highly resolved by phylogenetic inference and structure analysis.

Our analysis supports several key findings: Modern F_1_ hybrid Calabrese broccoli has undergone strong selective pressures and reduction in diversity compared to the open-pollinated landraces it was derived from. Morphologically, modern F_1_ hybrid Calabrese broccoli is distinct from its landrace predecessors, exhibiting accelerated maturity, more complete apical meristem arrest and dominance, higher harvest index, and superior heading quality characteristics. Several landrace accessions appear to be foundational as the initial source germplasm for modern hybrid Calabrese broccoli. While there are numerous signals of selection, several key genomic regions of reduced diversity and selective sweeps are particularly obvious with Calabrese broccoli hybrids and these regions harbor developmental candidates. Calabrese broccoli landraces are 4.8-fold richer in allelic diversity compared to Calabrese hybrids, and the larger pool of *italica* germplasm is more far rich in allelic diversity than is captured in modern hybrid Calabrese broccoli, and this diversity must be preserved as a resource for future broccoli and cauliflower improvement. There is not clear evidence that sprouting broccoli or violet cauliflower are the direct progenitor of Calabrese broccoli, or vice versa.

This work provides an overview of the genetic and morphological diversity in *B. oleracea* var. *italica* and clarifies the relationship of modern Calabrese broccoli hybrids with foundational germplasm via analysis of morphological changes, population differentiation, allelic diversity, selective sweeps, linkage disequilibrium, runs of homozygosity, and key population-specific SNPs. These results quantify the genetic erosion occurring in *italica* and underscores the importance of *in-situ* and *ex-situ* conservation efforts.

## Methods

### Genotyping

Leaf tissue from all entries were bulked from five plants at the 2–3 true leaf stage and extracted according to standard protocols^70^. Genotyping-by-sequencing (GBS) was accomplished at the University of Wisconsin Biotechnology Center DNA Sequencing Facility as previously described^71^. Libraries were construction in two 96-well plates using digestion by the restriction enzyme ApeKI and were sequenced on Illumina HiSeq 2500, producing 100-bp single-end reads. SNPs were produced using the TASSEL v5.2.57 GBS pipeline^72^. The raw sequence reads were aligned to the *B. oleracea* BOL.v2^6^ reference genome using Burrows-Wheeler Alignment (v.0.7.17) backtrack algorithm^73^. The TASSEL commands -DiscoverySNPCallerPluginV2 and -ProductionSNPCallerV2 were invoked with default parameters, followed by LD-KNNi imputation^74^ with the following parameters: high LD sites (30), number of nearest neighbors (10), max distance to find LD (10,000,000). Indels or sites assigned to unplaced scaffolds were removed, and one entry (B140) was removed from further analysis due to missing data, resulting in *N* = 109 entries. Sites with >10% missing data and minor allele frequencies (MAF < 0.05) were removed, resulting in 31,811 SNPs. GBS summary statistics by site, chromosome, taxa, and subpopulation were generated in TASSEL and visualized in R v3.6.1^75^. To identify subpopulation specific polymorphic alleles, the GBS data were divided by subpopulation for taxa assigned 50% membership to a given subpopulation in structure analysis and subsequently filtered for MAF > 5%, minor SNP states, and indels. Genome-wide SNP density was calculated by binning markers in 1 Mbp bins across physical locations. Linkage disequilibrium was calculated for all accessions and independently within subpopulations by estimating r^2^ for a given marker against all other markers within a 1 Mbp window using vcftools^76^ by invoking the –geno-r2 command. To compare linkage disequilibrium between subpopulations, distances in base pairs were log_10_ transformed and fit using a smoothed spline (spar = 0.5). Decay below the threshold (*r*^2^ < 0.2) was determined by choosing the smallest value of the smoothed spline falling below threshold values. For the Calabrese hybrids, the function estimate coefficients across all genomic regions were extracted and the top 1 percentile was selected. These regions were merged if overlap was less than 1 Mbp and intersected with the BOL.v2 annotation using the R package bedr v1.0.7^77^. Variant annotation was accomplished for all 31,811 markers using SnpEff v.4.3t^78^ using the BOL.v2 annotation under default settings.

### Diversity

PCA and multidimensional scaling using all markers and taxa was conducted in TASSEL by invoking the -PrincipalComponentsPlugin and -MultiDimensionalScalingPlugin, respectively. The PCA loadings explaining variance in PCA structure for each SNPs were calculated in TASSEL and the top 1% SNPs were filtered and intersected with the BOL.v2 annotation. Using all 31,811 markers and taxa (*N* = 109), a genetic similarity matrix was generated in PLINK (v1.90b6.15)^79^ by calculating the probability that randomly chosen alleles at a locus are identical by state (P_IBS_(AA,AA) = 1; P_IBS_(AA,BB) = 0, P_IBS_(AA,xx) = 0.5), averaged over all non-missing loci by invoking the command -distance with the ‘square’ and ‘ibs’ parameters. The similarity matrix was visualized using the heatmap2() function in R. A maximum likelihood based inference of phylogeny was conducted using the program RAxML (8.2.12)^80,81^ using the CIPRES Science Gateway server^82^ under the ML/thorough-bootstrap workflow, using the GTR + GAMMA bootstrapping model with one hundred alternative runs on distinct starting trees. The consensus tree was rooted using using the function root() in the R package ape^83^ and used to order the rows of the distance matrix and population structure analysis. For population structure analysis, the 31,811 SNPs were first pruned for linkage disequilibrium in PLINK using the function -indep-pairwise using a step size of 50 kb, a window size of 1 kb, and linkage disequilibrium threshold (*r*^2^ > 0.25). The Bayesian clustering algorithm structure.py was run across the values of K 2.20, assuming a simple prior in FastStruture (v.1.0)^84^. The FastStruture chooseK.py algorithm selected *K* = 4 as the optimal model complexity to explain the structure within the data as well as maximizing the marginal likelihood. All accessions were assigned to a subpopulation with a minimum threshold of 50% membership. Structure results were visualized using the R package popHelper^85^.

Genome-wide population differentiation (F_st_) was calculated when comparing the Calabrese hybrid subpopulation against all other subpopulations, using implementing the –weir-fst-pop function in vcftools to scan across all 31,811 markers in 1 Mbp windows with a 1 kbp step-size. Nucleotide diversity was estimated genome-wide by the sliding-window TASSEL plugin -diversitySlidingWinStep across 10 SNP markers using a 5 marker step size. Nucleotide diversity and reduced/enriched diversity were calculated as where x_i_ is the frequency of the ith sequence in the population and _pi j_ is the number of differences per nucleotide site between the ith and jth sequences^86^. ROD was calculated across 10 markers using a 5 marker step-width and the top 1 percentile genomic regions^5,87,88^ were reserved for further analysis. Selective sweep analysis was conducting using the Calabrese hybrid subpopulation with SweeD^89^. SweeD was run for each subpopulation by first disaggregating all 31,811 markers by chromosome and then adjusting the grid size to scan 1 kbp windows of each chromosome using a custom bash script. A screen for clustered runs of homozygousity were was conducted in PLINK using the –homozyg-group algorithm to scan across 1 Mbp windows, requiring a 0.99 or greater segment concordance between pairwise matches, allowing for one heterozygous call and five missing calls within windows. The top 1 percentile outliers for these regions were intersected with the BOLv.2 annotation using and a custom R script and the R package bedr that merged adjacent regions separated by less than 1 Mbp. These feature-rich genomic regions were searched against the online databases Ensembl Plants using the R package biomaRt^90,91^ and Tair^92,93^ and visualized using a custom R script.

### Germplasm and phenotyping

Hybrid F_1_ germplasm was gathered from multiple breeding programs and distributors (Supplementary Table 1). Landrace accessions collected in Italy typically contained geographical provenance, but if exact latitude and longitude coordinates were not supplied (e.g., an address), latitude and longitude was determined using Google Maps and mapped using the package maps^94^. Additional passport information including cultivar release year and breeding institution was collected from several sources^16,51,95^, and personal communication with breeders.

Lines were sown into 128 cell trays on 2019/05/05 (JD = 125) and 2019/06/03 (JD = 154) for plantings 1 and 2. Seedlings were grown in a greenhouse, transferred to cold frames after 4 weeks, and transplanted on JD 170 and JD 189 respectively into Lima silt loam fields in Geneva, NY (42.88 N, −77.03 W). All lines were randomized into three replications and transplanted onto raised beds with ~10 plants per genotype per plot, although some plots contained fewer plants due to seed low seed quantity or poor germination. Drip irrigation was applied as needed throughout the growing season and additional cultural practices were as previously described^28,52^. Plots were examined every other day and evaluated at heading maturity for heading traits, determined when between 1/3 to 2/3 of plants in a given plot had reached harvest stage.

Approximately 16 biennial or day length sensitive accessions that would normally form heading structures in cooler season Mediterranean climates did not head or flower and were excluded from evaluations. Traits within four trait classes were evaluated: architecture, biomass, head quality, and phenology^27^ (Supplementary Table 1 TRAITS). The traits (MT, HE, BR, BS, BU, HC, HU, and OQ) followed standardized protocol employed by the Eastern Broccoli Project^52^ using an ordinal scale (1 = worst; 5 = best). Plants were cut at ground level to evaluate BM, HT, WD, LT. Heads were trimmed 16 cm from crown apex, photographed, processed using the GNU Image Manipulation Program v2.10.8^96^ and visualized using a custom R script. The traits (HT, WD, LT, BM, HM, FC, FF, F50) were evaluated according to IBPGR standards^97^. The traits HD, CL, and RR were collected according to previously described protocols^98^. The phenology traits (MT, FF, F50) were calculated as days from sowing to maturity, first flowering, and 50% flowering respectively; holding ability (MT50) was calculated as F50 - MT. Summary statistics were calculated by summarizing genotypes across traits and environments. Spearman correlation coefficients were estimated using complete pairwise observations. Linear regression was conducted in R to compare horticultural quality traits with cultivar release year of relevant accessions and principal component axes. Relative importance analysis of horticultural quality was conducted using 1000 bootstrap replications of the “lmg” method in the R package RateRvaR (v. 1.0)^52,99^ using nonaveraged data by fitting the model: OQ ~ HT + WD + HE + LT + BM + HM + BS + BU + HC + HD + CL + RR + BR + HS + HU + HR + MT + FF + F50.

## Supplementary information

HORTRES-02970 Supplemental Tables

HORTRES-02970 Fig 1 high resolution

HORTRES-02970 Fig 4 high resolution

Dataset 3

Dataset 1

Dataset 2

## Data Availability

All data is included in supplementary materials or SRA:SUB7486659.

## Code availability

All code used in this analysis is available at https://github.com/zacharystansell/B_oleracea_diversity_panel.
