# Supplementary material for: From landrace to modern hybrid broccoli: the genomic and morphological domestication syndrome within a diverse B. oleracea collection
Source: Hortic Res. 2020 Oct 1;7:159. doi: 10.1038/s41438-020-00375-0 (PMC7528014; doi:10.1038/s41438-020-00375-0)
Supplement: Supplementary file 1 — HORTRES-02970 Supplemental Tables [file 41438_2020_375_MOESM1_ESM.docx]

**S. Table 1**: Accessions included in analyses, seed source, and accession collection location

| **Accession** | **Subpopulation** | **Source** | **Collection Location** |
| --- | --- | --- | --- |
| B001.Marathon | Calabrese.F1 | Sakata | - |
| B003.Diplomat | Calabrese.F1 | Sakata | - |
| B004.DuraPak19 | Calabrese.F1 | Syngenta | - |
| B011.Gypsy | Calabrese.F1 | Sakata | - |
| B013.Lieutenant | Calabrese.F1 | Seminis/Vanden Bosch/Royal Sluis | - |
| B014.Green.Magic | Calabrese.F1 | Sakata | - |
| B015.Brogan | Calabrese.F1 | Bejo | - |
| B016.Green.Harmony | Calabrese.F1 | Known You | - |
| B017.Major | Calabrese.F1 | Seminis/Vanden Bosch/Royal Sluis | - |
| B021.Liberty | Calabrese.F1 | Peto Seed | - |
| B047.Packman | Calabrese.F1 | Peto Seed | - |
| B093.Cavolo.Broccolo.Tardivo | Calabrese.LR | F. Branca/University of Catania | 41.2 x 16.3 |
| B094.Cavolo.Broccolo.Di.Sarno | Sprouting.Broccoli | F. Branca/University of Catania | 40.9 x 14.3 |
| B095.Verde.Calabrese.Precoce | Calabrese.LR | F. Branca/University of Catania | 41.9 x 12.5 |
| B096.Ramoso.Grande.Precoce | Calabrese.LR | F. Branca/University of Catania | 41.2 x 16.3 |
| B097.Ramoso.Calabrese | Calabrese.LR | F. Branca/University of Catania | - |
| B098.Cavolo.Broccolo.Natalina | Violet.Caul | F. Branca/University of Catania | 38.1 x 13.3 |
| B099.Cavolo.Broccolo.Frevarota | Violet.Caul | F. Branca/University of Catania | 38.1 x 13.3 |
| B100.Cavolo.Broccolo.Marzullo | Violet.Caul | F. Branca/University of Catania | 38.1 x 13.3 |
| B101.Cavolo.Broccolo.Invernale.Type.A | Sprouting.Broccoli | F. Branca/University of Catania | 38.2 x 15.6 |
| B102.Ramoso.Calabrese | Calabrese.LR | F. Branca/University of Catania | 44.8 x 10.3 |
| B103.Broccolo.Verde.Di.Calabria.Precocissimo | Calabrese.LR | F. Branca/University of Catania | 40.6 x 14.9 |
| B104.Cavolo.Zolfino | Sprouting.Broccoli | F. Branca/University of Catania | - |
| B105.Precoce.Di.Calabria.Tipo.Esportazione | Calabrese.LR | F. Branca/University of Catania | 40.6 x 14.9 |
| B106.Picolini.Di.Palermo | Sprouting.Broccoli | F. Branca/University of Catania | 38.1 x 13.3 |
| B107.Ramoso.Calabria.Mezzo.Precoce | Calabrese.LR | F. Branca/University of Catania | 45.4 x 11.9 |
| B108.Verde.Calabrese | Calabrese.LR | F. Branca/University of Catania | - |
| B109.Ramoso.Calabrese.Verde | Calabrese.LR | F. Branca/University of Catania | 41.1 x 16.9 |
| B110.Verde.Calabrese | Calabrese.LR | F. Branca/University of Catania | 41.1 x 16.9 |
| B111.Natalino.Di.Napoli | Sprouting.Broccoli | F. Branca/University of Catania | 41.1 x 16.9 |
| B112.Cavolo.Broccolo.Settembrina | Violet.Caul | F. Branca/University of Catania | 38.1 x 13.3 |
| B113.Broccolo.Innorota.Gennorota | Violet.Caul | F. Branca/University of Catania | 38.1 x 13.3 |
| B114.Cavolo.Broccolo | Sprouting.Broccoli | F. Branca/University of Catania | 38.2 x 13.3 |
| B115.Broccolo.Ramoso.Di.Calabria | Calabrese.LR | F. Branca/University of Catania | - |
| B116.Broccolo | Sprouting.Broccoli | F. Branca/University of Catania | 38.6 x 16.2 |
| B117.Natalisa.Ciurietti | Violet.Caul | F. Branca/University of Catania | 36.9 x 14.8 |
| B118.Broccolo.Destate | Sprouting.Broccoli | F. Branca/University of Catania | 37.5 x 15.1 |
| B119.Broccolo.Natalino | Sprouting.Broccoli | F. Branca/University of Catania | 37.5 x 15.1 |
| B120.Broccolo.Apriloto | Sprouting.Broccoli | F. Branca/University of Catania | 37.5 x 15.1 |
| B121.Maialora | Violet.Caul | F. Branca/University of Catania | 36.9 x 14.8 |
| B122.Ramoso.Calabrese | Calabrese.LR | F. Branca/University of Catania | 41.2 x 16.3 |
| B123.Ramoso.Calabrese | Calabrese.LR | F. Branca/University of Catania | 40.8 x 14.3 |
| B124.Ramoso.Calabrese.Precoce | Calabrese.LR | F. Branca/University of Catania | 42.5 x 14.2 |
| B125.Ramoso.Atlantic.Local | Calabrese.LR | F. Branca/University of Catania | 42 x 15 |
| B126.Ramoso.Calabrese | Calabrese.LR | F. Branca/University of Catania | 43.6 x 13.5 |
| B127.Ramoso.Calabrese.Precoce | Sprouting.Broccoli | F. Branca/University of Catania | 40.9 x 14.3 |
| B128.Broccolo.Natalino.Di.Sarno | Sprouting.Broccoli | F. Branca/University of Catania | 40.9 x 14.3 |
| B129.Ramoso.Calabria.Tardivo | Calabrese.LR | F. Branca/University of Catania | 45.4 x 11.9 |
| B130.Cavolo.Broccolo.Agostina | Violet.Caul | F. Branca/University of Catania | 38.1 x 13.3 |
| B131.Broccolo.Verde.Di.Calabria.Tardivo | Calabrese.LR | F. Branca/University of Catania | 40.6 x 14.9 |
| B132.Precoce.Di.Calabria | Calabrese.LR | F. Branca/University of Catania | 40.6 x 14.9 |
| B133.Ramoso.Calabrese | Calabrese.LR | F. Branca/University of Catania | 39.3 x 16.3 |
| B134.Cavolo.Sparacello | Sprouting.Broccoli | F. Branca/University of Catania | 37.3 x 13.6 |
| B135.N.tagliu.N.tagghiv.Pilotu | Sprouting.Broccoli | F. Branca/University of Catania | 37.7 x 14.8 |
| B136.Ramoso.Calabrese.Verde | Calabrese.LR | F. Branca/University of Catania | 41.7 x 15.4 |
| B137.Ramoso.Calabrese.Precoce | Calabrese.LR | F. Branca/University of Catania | 41.2 x 16.3 |
| B138.Broccolo.No.1 | Sprouting.Broccoli | F. Branca/University of Catania | 39.1 x 16.5 |
| B139.Di.Sicilia.Catanese | Violet.Caul | F. Branca/University of Catania | 45.5 x 9.2 |
| B141.BH028 | Calabrese.F1 | M. Farnham/USDA-ARS | - |
| B142.BH034 | Calabrese.F1 | M. Farnham/USDA-ARS | - |
| B143.BH027 | Calabrese.F1 | M. Farnham/USDA-ARS | - |
| B144.BC1691 | Calabrese.F1 | Seminis/Vanden Bosch/Royal Sluis | - |
| B145.BC1764 | Calabrese.F1 | Seminis/Vanden Bosch/Royal Sluis | - |
| B146.Montvert | Calabrese.F1 | Syngenta | - |
| B147.DuraPak16 | Calabrese.F1 | Syngenta | - |
| B148.Burney | Calabrese.F1 | Bejo | - |
| B150.Eastern.Crown | Calabrese.F1 | Sakata | - |
| B151.Imperial | Calabrese.F1 | Sakata | - |
| B152.Emerald.Crown | Calabrese.F1 | Sakata | - |
| B153.Everest | Calabrese.F1 | Sakata | - |
| B154.BH019 | Calabrese.F1 | M. Farnham/USDA-ARS | - |
| B155.BH020 | Calabrese.F1 | M. Farnham/USDA-ARS | - |
| B156.BH026 | Calabrese.F1 | M. Farnham/USDA-ARS | - |
| B159.BH044 | Calabrese.F1 | M. Farnham/USDA-ARS | - |
| B181.Green.Numen | Calabrese.F1 | Evergrow | - |
| B182.Green.Elegance | Calabrese.F1 | Evergrow | - |
| B187.Clara | Calabrese.F1 | Known You | - |
| B189.EMBR934 | Calabrese.F1 | Emerald | - |
| B190.EMBR958 | Calabrese.F1 | Emerald | - |
| B191.Waltham29 | Calabrese.LR | High Mowing | - |
| B192.Premium.Crop | Calabrese.F1 | Takii | - |
| B193.TBR130 | Calabrese.F1 | Tainong | - |
| B194.Corato | Calabrese.F1 | Enza-Zaden | - |
| B195.Belstar | Calabrese.F1 | Bejo | - |
| B196.Power.Dome | Calabrese.F1 | Tainong | - |
| B198.Fiesta | Calabrese.F1 | Bejo | - |
| B200.Blue.Wind | Calabrese.F1 | Johnnys | - |
| B203.Calabrese | Calabrese.LR | SSE | - |
| B204.Cavolo.Broccoli.Ramoso.Calabrese | Calabrese.LR | Franchi/SoI | - |
| B205.Cavolo.Broccoli.Ramoso.Calabrese | Calabrese.LR | Franchi/SoI | - |
| B206.Covina | Calabrese.F1 | Bejo | - |
| B210.Cavolfiore.Romanesco.Precoce | Sprouting.Broccoli | Franchi/SoI | - |
| B211.Gianfranco.Fuscello.Spiagariello.Lisco | Sprouting.Broccoli | Franchi/SoI | - |
| B212.Cavolo.Broccolo.Spigariello | Sprouting.Broccoli | Franchi/SoI | - |
| B213.Atlantis | Calabrese.F1 | Johnnys | - |
| B214.Athlete | Calabrese.F1 | NE seed | - |
| B215.Monflor | Calabrese.F1 | Syngenta | - |
| B216.Burgundy | Violet.Caul | Johnnys | - |
| B217.Millenium | Calabrese.F1 | Sakata | - |
| B218.Ironman | Calabrese.F1 | Seminis/Vanden Bosch/Royal Sluis | - |
| B219.Emerald.Jewel | Calabrese.F1 | Sakata | - |
| B221.Heritage | Calabrese.F1 | Seminis/Vanden Bosch/Royal Sluis | - |
| B222.Baccus | Calabrese.F1 | Asgrow | - |
| B223.Sabre | Calabrese.F1 | Asgrow | - |
| B224.Saga | Calabrese.F1 | Johnnys | - |
| B225.Coronado.Crown | Calabrese.F1 | Seminis/Vanden Bosch/Royal Sluis | - |
| B226.General | Calabrese.F1 | Seminis/Vanden Bosch/Royal Sluis | - |
| B227.Asteroid | Calabrese.F1 | Harris Moran | - |
| B261.DeCicco | Calabrese.LR | Johnnys | - |

**S. Table 2:** Traits evaluated, by code, trait class, and description.

| **Code** | **Class** | **Description** |
| --- | --- | --- |
| HT | architecture | Plant height: ground to apex (cm) |
| WD | architecture | Plant width: max leaf distance (cm) |
| HE | architecture | Head extension (1 = low; 5 = high) |
| LT | architecture | Lateral shoots (count) |
| BM | biomass | Above ground biomass (g) |
| HM | biomass | Head mass, trimmed to 16 cm (g) |
| HI | biomass | Harvest Index; (HM/BM) |
| BS | head quality | Mean bud diameter (N=5) |
| BU | head quality | Bead Uniformity ( 1 = not uniform; 5 = very uniform) |
| HC | head quality | Head Compactness (1 = loose; 5 = very firm) |
| HD | head quality | Max Head Diameter head diameter (cm) |
| CL | head quality | Max cluster diameter (cm) |
| RR | head quality | First rank branching length (cm) |
| BR | head quality | Bracting (1= extreme ; 5 = no bracting) |
| HS | head quality | Head Shape (1 = flat; 5 = very convex) |
| HU | head quality | Head Uniformity (1 = not uniform; 5 = very uniform) |
| HR | head quality | Heat Response (1 = severe heat response; 5 = no heat response) |
| OQ | head quality | Overall Quality (1= not recognizable as broccoli ;  5 = very high quality) |
| FC | head quality | Flower Color (1=white; 0=yellow) |
| MT | phenology | Days to plot maturity (d) |
| FF | phenology | Days to first flowering (d) |
| F50 | phenology | Days to 50% flowering (d) |
| MF50 | phenology | Holding ability; F50 – HM (d) |
