# Supplementary figures and images for: From landrace to modern hybrid broccoli: the genomic and morphological domestication syndrome within a diverse B. oleracea collection
Source: Hortic Res. 2020 Oct 1;7:159. doi: 10.1038/s41438-020-00375-0 (PMC7528014; doi:10.1038/s41438-020-00375-0)

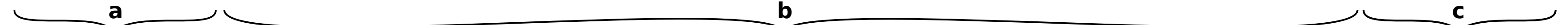

Supplement: Supplementary file 3 — HORTRES-02970 Fig 4 high resolution [file 41438_2020_375_MOESM3_ESM.pdf]
